# A case report of a chronic migraine patient treated with three different anti-CGRP monoclonal antibodies: which parameters better represent the efficacy?

**DOI:** 10.3389/fneur.2023.1176816

**Published:** 2023-05-05

**Authors:** Sena Uzun, Ulf Frejvall, Gülsen Özkaya-Sahin, Gürdal Sahin

**Affiliations:** ^1^SkåNeuro Neurology Clinic, Lund, Sweden; ^2^Department of Clinical Sciences of Malmö and Lund, Lund University, Lund, Sweden; ^3^Department of Laboratory Medicine, Section of Clinical Microbiology, Lund, Sweden; ^4^Department of Translational Medicine, Lund University, Malmö, Sweden

**Keywords:** anti-CGRP monoclonal antibodies, chronic migraine, erenumab, fremanezumab, galcanezumab, case report

## Abstract

**Objective:**

To report the efficacy of different anti-calcitonin gene-related peptide (CGRP) monoclonal antibodies (mAbs) on headache frequency, intensity, and duration.

**Background:**

Blockade of CGRP receptors or neuropeptide with anti-CGRP mAbs have been successfully used for several years for the prevention of chronic and episodic migraine. The response is usually assessed by improvement seen in the number of days with headache per month. However, clinical praxis indicates that sole reliance on the frequency of headaches might be insufficient to interpret the efficacy of these treatments.

**Methods:**

Retrospective review of a case with a meticulous headache diary who has tried three different anti-CGRP mAbs for chronic migraine prevention.

**Results:**

The patient has been diagnosed with chronic migraine and was first treated with erenumab, followed by fremanezumab and thereafter galcanezumab due to several reasons. In addition to significant improvement in all three parameters analyzed with anti-CGRP mAb treatment, the most important and valuable effect on the patient's quality of life was decreased duration and frequency of headaches. At present, the patient is receiving fremanezumab treatment with an excellent tolerability.

**Conclusion:**

There is a clear need for careful follow-up and detailed daily records of headaches showing the frequency, duration, and severity for the evaluation of anti-CGRP mAbs treatment. This study shows the importance of this information in order for medical professionals to make an informed decision regarding the best course of anti-CGRP mAbs treatment in cases of side effects or lack of efficacy.

## Introduction

Chronic migraine (CM) is a neurological disease characterized by more than 15 headache days per month with at least 8 days of migraine for more than a period of 3 months according to the criteria of the third edition of the International Classification of Headache Disorders (ICHD-3) (1). CM is one of the most common causes of disability and affects 1–2% of the population worldwide (2). The vast majority of patients require preventive therapies to sustain a reasonable quality of life (3). Anti-calcitonin gene-related peptide (CGRP) monoclonal antibodies (mAbs) either targeting the CGRP receptor (erenumab) or the CGRP neuropeptide (fremanezumab, galcanezumab, and eptinezumab) have been used successfully to prevent migraine attacks in patients who have not responded well to standard treatment for several years (4). Yet, the availability of anti-CGRP mAbs differs across the world. Although head-to-head studies are lacking, they seem to have comparable efficacy and favorable side effect profile. The response is typically measured by the improvement in the monthly headache days and reimbursement agencies usually have the requirement of a minimum 25–30% reduction (5–7). However, to identify therapy benefits accurately it may be advantageous to include other parameters, e.g., headache duration and intensity. Here in this paper, we present a case with CM who has a habit of using a headache diary dedicatedly and diligently with records of frequency, duration, and intensity. This patient had also used three different anti-CGRP mAbs across a 36-month period.

## Clinical case

**Figure 1:**
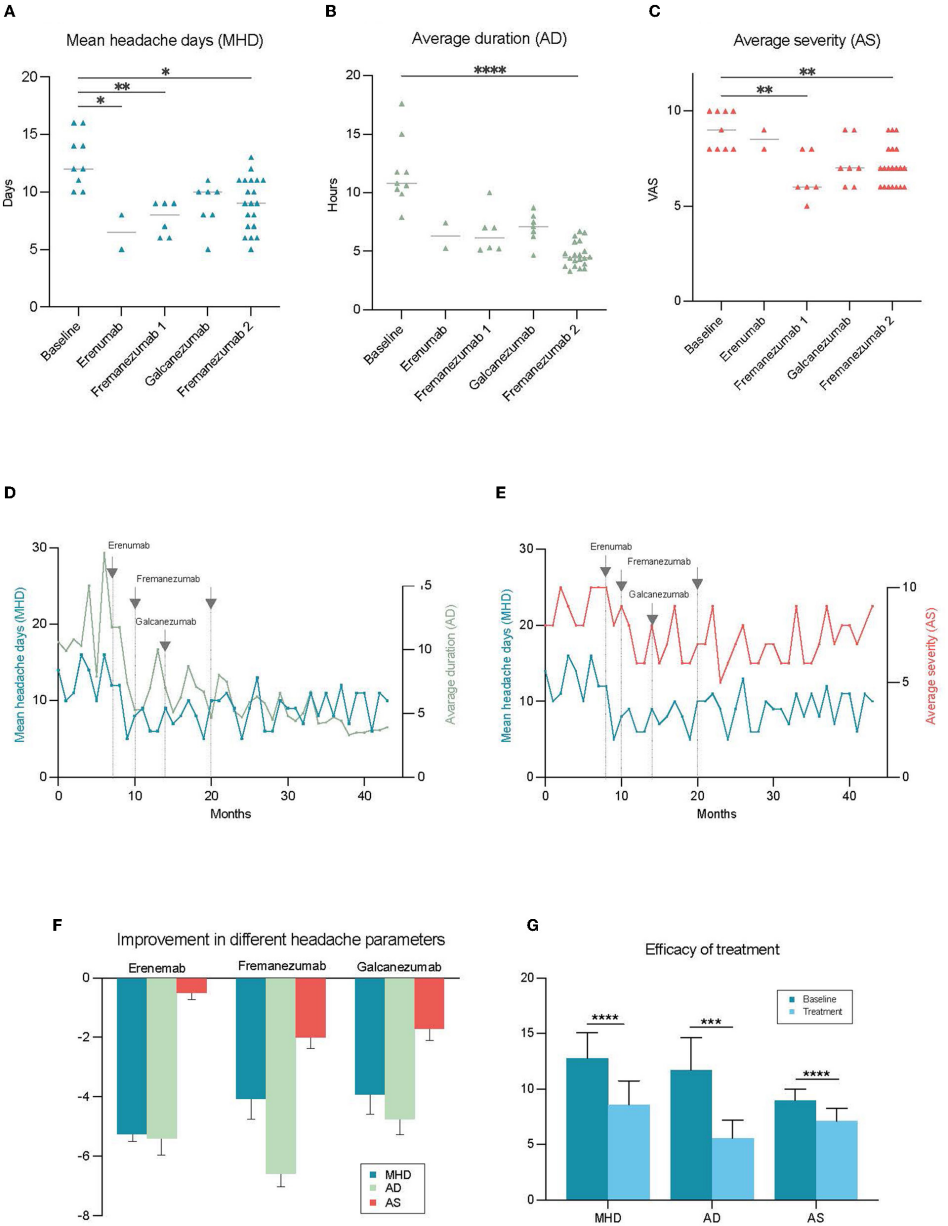
Improvement in the different headache parameters during treatment with anti-CGRP mAbs. Each point shows the value of the chosen parameter by month. MHD was reduced significantly with erenumab and fremanezumab **(A)**, and decrease in AD and AS was significant only with the fremanezumab treatment **(B, C)**. The amplitude of change was highest in AD among all parameters and reported to be the most impactful for the patient **(D, E)**. **(F)** Illustrates the differential changes in various headache parameters in response to different treatments. The overall efficacy of anti-CGRP mAb treatments was significant for each parameter. For MHD mean difference was 4.1**** days, AD 6.2 *** hours, AS 1.9 *** **(G)**. *p < 0.05, **p < 0.01, ***p < 0.001, ****p < 0.0001 (AD, average duration of headache; AS, average severity of headache; MHD, mean headache days per month).

A 45 year old woman was referred to our clinic in October 2019 with treatment-resistant migraine. She had headache since childhood and, migraine with one-sided, alternating, throbbing headaches localizing generally on her forehead and sometimes radiating to the neck since she was 22 years old. The migraine became frequent after she was 34 years old. Headache duration was often up to 48–72 h per occasion. Yawning was experienced in the prodromal phase. She never had an aura. During the attacks, she presented with both photophobia and phonophobia, with nausea usually commencing afterward. She has seldom had vomiting. Standard therapies, sumatriptan and ibuprofen, failed to control these attacks. She had 13.0 ± 2.3 (SD) monthly headache days (MHD) on average during 3 months before treatment. The average duration (AD) was 12.0 ± 2.9 h and average severity (AS) was 9.0 ± 1.0 from 10 according to the visual analog scale (VAS) per occasion before the treatment (8) (see Figures 1A–C). Her blood pressure was 120/69 mmHg and pulse rate were 51 bpm. Neurological examination was unremarkable. She had an ependymoma in the fourth ventricle and this was removed by the age of 30. She thinks that the brain surgery was critical in her migraine history, as it made her neck muscles weak.

CM diagnosis was confirmed on the basis of having more than 15 headache days in at least 3 of the last 12 months according to ICHD-3 (1). The patient was given erenumab 70 mg since she had tried metoprolol, verapamil, and topiramate as preventive treatment previously. The MHD, AD, and AS were respectively 6.5 ± 2.1 days, 8.9 ± 1.5 h, and 8.5 ± 0.7 after two months of erenumab treatment. Despite this satisfactory improvement, erenumab was switched to fremanezumab 675 mg because of the side effect of constipation. She also switched her acute medication from sumatriptan 25 mg tablet to zolmitriptan 2.5 mg nasal spray. Fremanezumab was used for 6 months without any side effects. During this period of fremanezumab treatment, MHD was 7.4 ± 1.5 days, AD was 6.7 ± 1.8 h, and AS was 6.8 ± 1.2. All three parameters showed a statistically significant improvement compared to the baseline (Figures 1A–C). She had to switch her preventive treatment to galcanezumab 120 mg since she was living transiently in Australia where fremanezumab was unavailable at that time. Galcanezumab was used for 7 months with an inferior efficacy profile [MHD (8.9 ± 2.0 days), AD (7.0 ± 1.3 h), and AS (7.3 ± 1.2)] compared to fremanezumab, hence she switched to fremanezumab 225 mg when she moved back to Sweden (see Figures 1A–C). She describes the reduction in the duration of headaches as the most critical factor improving her quality of life. Currently, she is under treatment with fremanezumab for 21 months, and the efficacy particularly with regards to duration and intensity of the headaches has been improving over time. According to the notes from her last visit to the clinic, MHD, AD, and AS are 8.9 ± 2.2 days, 4.8 ± 1.0 h, and 6.4 ± 1.0 respectively since she was on fremanezumab (see Figures 1D, E for change in headache parameters over time).

For statistical comparisons between unrelated numerical variables, MHD, AD, and AS, Mann-Whitney *U* test, or Kruskal Wallis test were used as appropriate. Analyses were performed using GraphPad Prism 9.5.1 and significance was accepted at *p* < 0.05.

This study was approved by the Swedish Ethical Review Authority with a diary number of 2022-05183-01 and the patient has signed written informed consent.

## Discussion

We present a patient with CM who has kept track of migraine in detail with headache days, duration, and intensity over the years which gave us the possibility to comprehensively compare the efficacy of preventive treatments. She has switched between three different anti-CGRP mAbs because of side effects, availability issues with the aim for better efficacy.

This case shows us a clear discrepancy between the different features of headache in response to preventive treatment with anti-CGRP mAbs (see Figure 1F). Although the efficacy was modest in MHD and AS, the patient has experienced a revolutionary effect in AD decreasing from 12.0 to 4.8 h (see Figure 1G). She mentions this as the most impactful change that significantly improved her quality of life. On days when she wakes up with a migraine attack, 5 h following treatment with acute treatment, she reports that she was able to continue her day as normal. What also has been fundamental is that she no longer gets the most severe peaks in pain. Moreover, we believe that this case is instructive by underlining differential side effects and efficacy profiles between the different anti-CGRP mAbs which demonstrates the importance of switching to another anti-CGRP mAbs when necessary. There is an increasing amount of awareness in the literature supporting the abovementioned need of using additional parameters when analyzing the efficacy of the treatment. Different headache features such as severity and duration are suggested as important secondary outcomes for both episodic and chronic migraine (9, 10).

In addition to being a single case report, there are several limitations that should be considered when interpretating the treatment outcome in this patient. Firstly, different treatment durations e.g., 2 months with erenumab, 7 months with galcanezumab, and 27 months with fremanezumab make it difficult to compare the precise efficacy. This makes the choice of statistical tests difficult. One could argue that the data points should be compared as related however we believe that unrelated comparison gives the best possible way to represent the efficacy of each antibody compared to the baseline features. Secondly, the treatment response could change over the years while the individuals with migraine improve and/or adjust their lifestyle in the presence of efficient treatment and this could lead to an overestimation of efficacy during fremanezumab treatment as this drug was the latest and longest prescribed treatment for the patient. Nevertheless, this case report still demonstrates the importance of comparison between the different available treatments and altering treatment plans for the patient when necessary to avoid side effects.

## Conclusion

In conclusion, this case represents us importance of evaluation of duration and severity of the headaches in addition to the frequency especially for the patients having modest efficacy on the treatment. Having such a detailed headache diary could help managing the migraine better to find the optimal treatment option. At present, there are no guidelines for which anti-CGRP mAbs we should prescribe and when to switch treatments. However, there is emerging consensus in the medical community to prescribe another anti-CGRP mAbs when faced with unacceptable side effects or lack of efficacy (4). Although most health agencies require a substantial decrease in headache days for reimbursement purposes, we believe that headache duration and severity are also critical parameters to consider.

## Data availability statement

The original contributions presented in the study are included in the article/supplementary material, further inquiries can be directed to the corresponding author.

## Ethics statement

Written informed consent was obtained from the patient for the publication of this case report.

## Author contributions

All authors listed have made a substantial, direct, and intellectual contribution to the work and approved it for publication.
